# Understanding the Risk Factors, Burden, and Interventions for Chronic Respiratory Diseases in Low- and Middle-Income Countries: A Scoping Review

**DOI:** 10.3389/phrs.2024.1607339

**Published:** 2024-10-31

**Authors:** Perla Boutros, Nour Kassem, Valentin Boudo, Ali Sié, Stephen Munga, Martina A. Maggioni, Marcin Golec, Robin Simion, Till Bärnighausen, Volker Winkler, Sandra Barteit

**Affiliations:** ^1^ Heidelberg Institute of Global Health, Heidelberg University Hospital, Heidelberg, Germany; ^2^ Centre de Recherche en Santé de Nouna, Nouna, Burkina Faso; ^3^ Kenya Medical Research Institute (KEMRI), Nairobi, Kenya; ^4^ Institute of Physiology, Center for Space Medicine and Extreme Environment, Charité Universitätsmedizin Berlin, Berlin, Germany; ^5^ Department of Biomedical Sciences for Health, Faculty of Medicine and Surgery, University of Milan, Milan, Italy; ^6^ Charité Center for Global Health, Charité Universitätsmedizin Berlin, Berlin, Germany; ^7^ Africa Health Research Institute (AHRI), Durban, South Africa; ^8^ Department of Global Health and Population, School of Public Health, Harvard University, Boston, MA, United States

**Keywords:** COPD, asthma, low-and middle-income countries, sub-Saharan Africa, climate change

## Abstract

**Objective:**

This scoping review aims to identify risk factors for COPD and asthma, examine the burden and intervention measures, and clarify the findings in the context of climate change, with a particular focus on LMICs.

**Methods:**

Following the PRISMA-ScR guidelines, we conducted a scoping review using PubMed, Embase, and Scopus, focusing on studies published from 2011 to 2024.

**Results:**

Our review included 52 studies that encompassed 244,004 participants. Predominantly conducted in SSA (n = 43, 83%) and Asia (n = 16, 31%), they address indoor and ambient air pollution, occupational hazards, and environmental conditions. Climate change exacerbates risks, varying regionally. SSA faces severe household and occupational exposures, while other LMICs deal with industrial and urban pollution. Stigma, social exclusion and economic burden underscore the necessity for intervention strategies (e.g., educational programs, pulmonary rehabilitation, low-emission cookstoves).

**Conclusion:**

Our research shows a strong link between air pollution, occupational and environmental exposures, and the prevalence of COPD and asthma in LMICs. It suggests that targeted interventions are effective ways to mitigate these diseases and also highlights the significant impact of climate change on respiratory health.

## Introduction

Chronic respiratory diseases like chronic obstructive pulmonary disease (COPD) and asthma have a significant impact on low- and middle-income countries (LMICs) due to environmental and socio-economic factors. Globally, COPD affects 4.1% of the population, while asthma affects 3.7% [1]. Asthma is characterized by chronic lower respiratory inflammation triggered by various factors like smoke, pollutants, and allergens [2], while COPD involves irreversible airway obstruction from chronic inflammation [3]. In 2019, out of the 391.9 million individuals aged 30–79 years affected by COPD worldwide, 315.5 million resided in LMICs [4]. Furthermore, LMICs account for over 80% of asthma-related deaths [3] and 90% of COPD-related deaths [5].

In LMICs, COPD accounts for approximately 1,000 disability-adjusted life years (DALYs) per 100,000 individuals [6]. In Sub-Saharan Africa (SSA), the rate is slightly lower at 715 DALYs per 100,000 individuals [7]. Furthermore, both COPD and asthma are often underserved diseases, exacerbated by the limited availability and cost-effectiveness of asthma medications and diagnostic procedures [3]. Limited access to healthcare, exposure to pollutants, and inadequate public health policies exacerbate these conditions in LMICs [3]. Asthma medications like long-acting muscarinic antagonists (LAMAs) are often scarce and costly in LMICs, impacting treatment availability [8–10]. Prioritizing preventive interventions, early diagnosis, and improving access to essential medications is crucial in reducing the burden of these diseases in LMICs [11].

In addition to the high prevalence, climate change, characterized by rising temperatures, increased heat exposure, and varying humidity levels, is emerging as a potentially under-explored risk factor that exacerbates respiratory diseases [12]. Elevated temperatures and weather variability, linked to climate change, may increase the frequency and concentration of airborne pollutants, allergens, and ozone, potentially worsening respiratory conditions [13]. Additionally, climate change-induced weather extremes may increase dust exposure [14], whereby inhaling dust particles may cause or worsen inflammation and irritation in the airways, leading to acute respiratory reactions such as bronchoconstriction, excess mucus production, and increased inflammation. Dust often contains allergens and microbes, introducing further respiratory irritants [15]. High humidity can increase airway resistance because of its high water content and raise the physiological demand for oxygen, resulting in heightened breathlessness [16]. Additionally, high humidity may promote mold growth, posing an additional risk to both healthy individuals and those with asthma and COPD who already have sensitive airways [17]. The World Meteorological Organization (WMO) predicts a 66% chance of global temperatures rising by at least 1.5° above pre-industrial levels between 2023 and 2027, potentially leading to a significant increase in the exacerbation of respiratory diseases [18]. SSA is already established as a highly vulnerable region to the impacts of climate change and extreme weather events [19, 20], experiencing the sharpest increases in temperature and changes in rainfall patterns [21].

However, research on obstructive lung diseases, notably COPD and asthma, is both limited and unevenly distributed worldwide [22]. In the literature, we identified five systematic reviews focusing on COPD in SSA [23–27], primarily examining the prevalence of the disease [23, 25–27], inadequate supply and quality of spirometry tests and equipment [23, 24, 27], risk factors [26], and the lack of non-communicable disease (NCD) healthcare plans [23, 24]. These studies primarily focused on countries such as South Africa, Nigeria, Tanzania, Malawi, Uganda, and Ethiopia, leaving many other low- and middle-income countries (LMICs) unexplored. Asthma research in SSA is scant and primarily centered on South Africa, while studies from high-income countries often lack relevance for SSA and other LMICs due to distinct population and environmental factors [26, 28, 29]. Key knowledge gaps regarding the impact of various risk factors and climate change on the health of individuals with COPD and asthma, as well as the evaluation of the effectiveness of current interventions, remain unexplored in LMICs.

To address these knowledge gaps, we conducted a scoping review and interpreted the findings in the context of climate change. This review was guided by the following research objectives:

1. To identify major risk factors of obstructive lung diseases, specifically COPD and asthma,
2. To elucidate the burden of disease of living with COPD and asthma and,
3. To determine intervention approaches and their effectiveness in managing and preventing COPD and asthma

## Methods

The scoping review adhered to the methodological framework by Arksey and O’Malley [30], refined by Levac et al. [31], following defined steps like defining research questions, selecting studies, charting data, and reporting findings in line with Preferred Reporting Items for Systematic Reviews and Meta Analyses for Scoping Reviews (PRISMA-ScR) guidelines [32].

### Inclusion and Exclusion Criteria

The study employed the Population-Exposure-Outcome (PEO) framework (see Supplementary Table S1), refining screening criteria and focusing on English primary research articles post-1st January 2011. This aligns with van Gemert et al.’s seminal work in 2011, evaluating asthma and COPD risk factors, impacts in SSA, and suggesting intervention strategies [26].

Our review encompassed studies from LMICs [33] exploring COPD and asthma as exposures, assessing risk factors, interventions for prevention and management, and challenges faced by affected individuals. Excluded were studies solely focusing on disease prevalence.

### Search Strategy

We searched electronic databases, including PubMed, Embase, and Scopus. Additionally, we examined grey literature via Google Scholar, focusing on the first 200 search results. Reference lists of relevant articles were hand-searched to manually identify and include relevant studies. Our search strategy incorporated index terms (e.g., MeSH, Emtree) and relevant keywords specific to the topic and context (see Supplementary Table S2).

### Study Selection

Articles identified from the databases were imported into Rayyan software [34], which removed duplicates. Two independent reviewers (PB, NK) evaluated the studies in a two-step process, using the predefined inclusion and exclusion criteria. Initially, titles and abstracts were screened. If either reviewer deemed a study potentially relevant, a full-text review was undertaken. A study was excluded only if both reviewers agreed on its irrelevance.

### Data Charting

Relevant data were extracted and organized in a Microsoft Excel spreadsheet. The charting process included parameters of title, author, publication year, country of origin, study objectives, target population, sample size (if provided), research design, analytical methods, gender distribution, age range, and primary findings.

### Reporting the Results

A narrative synthesis was conducted to provide an overview of the primary emerging themes from the included studies.

## Results

### Overview

**FIGURE 1:**
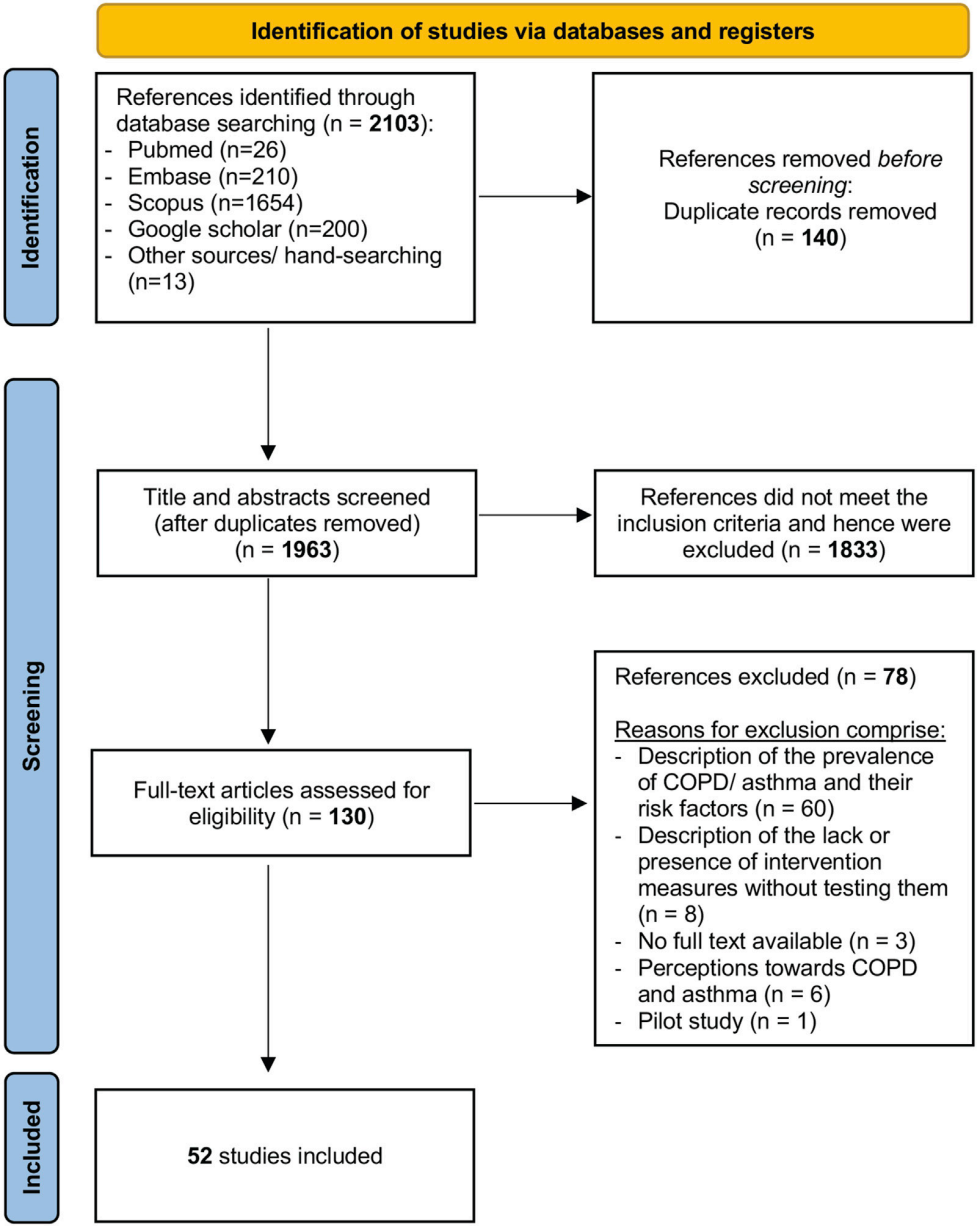
Preferred Reporting Items for Systematic Reviews and Meta-Analyses extension for Scoping Reviews flowchart (scoping review, low- and middle-income countries, 2022–2024).

The initial database search was conducted on August 9th, 2022, and updated on 4th January 2024. The results of the latter search are presented in the PRISMA-ScR flowchart (refer to Figure 1). After deduplication and incorporating 13 manually searched references from relevant studies, a total of 2,103 studies were considered. After screening titles, abstracts and full texts, n = 130 full-text articles were reviewed, of which n = 52 studies met the inclusion criteria and were subsequently included into the scoping review.

### Study Characteristics

**FIGURE 2:**
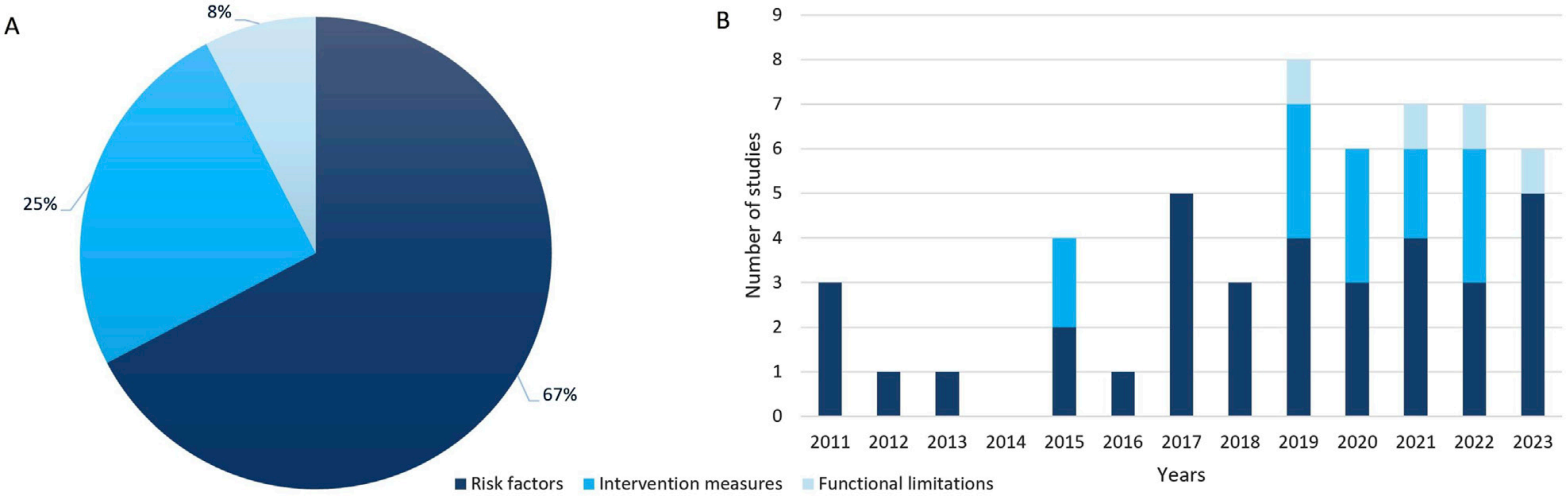
Distribution **(A)** and trend **(B)** of publications in low- and middle-income countries from 2011 to 2023 highlighting key areas: risk factors, intervention measures and functional limitations (scoping review, low- and middle-income countries, 2022–2024).

**FIGURE 3:**
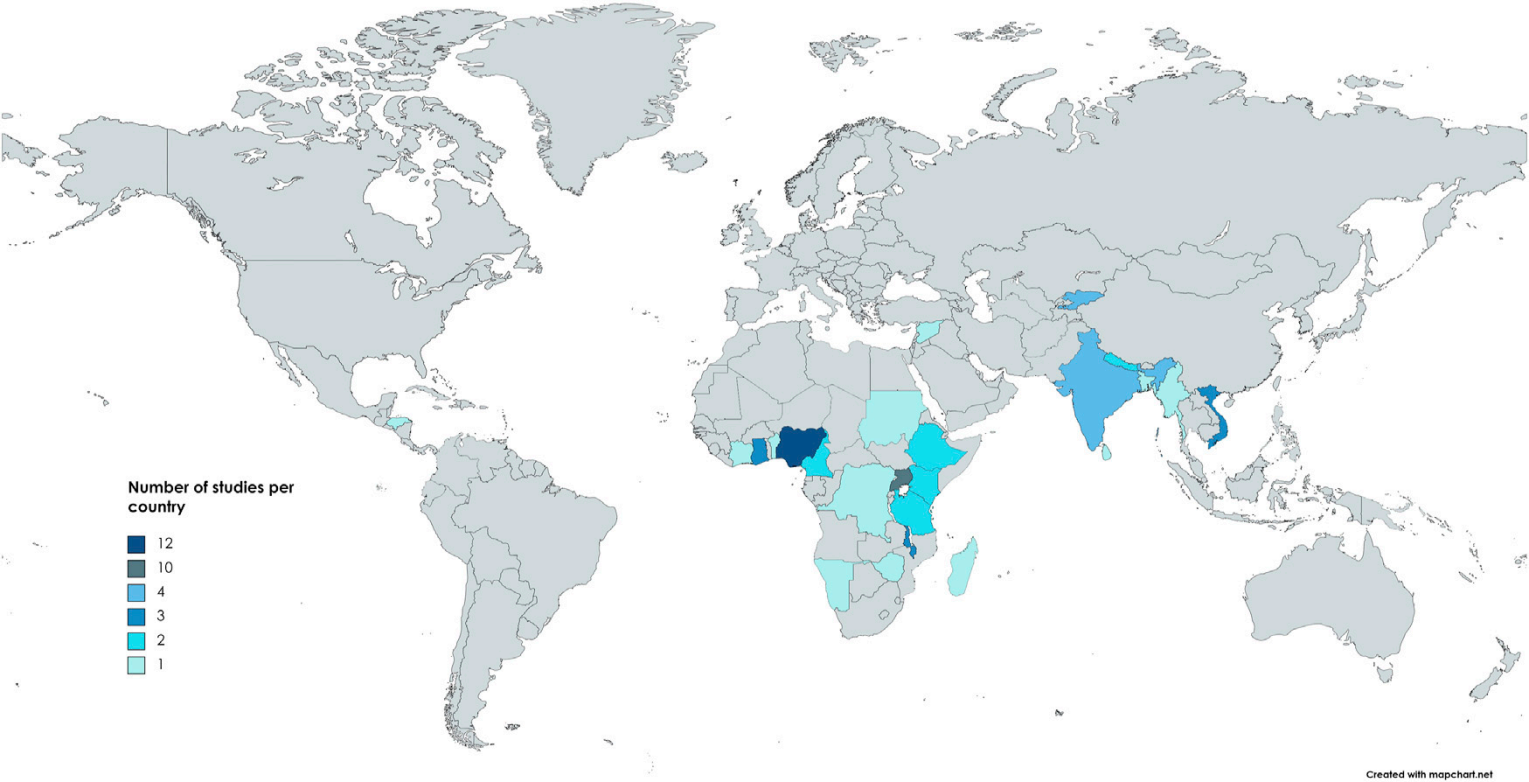
Distribution of included studies per country (scoping review, low- and middle-income countries, 2022–2024).

In this review, we analyzed data from a total study population of n = 244,004 participants covered in n = 52 studies (see Supplementary Table S3). The largest study, conducted in India in 2012, included n = 156,316 (64%) participants. From 2011 to 2022, publications on interventions for obstructive lung diseases in LMICs (see Figure 2) increased. Six studies (12%) were multinational in scope [35–40]. Specifically, n = 43 studies (83%) were conducted in SSA, representing n = 76,481 participants (31%), with Nigeria and Uganda contributing twelve (23%) and ten (19%) studies, respectively. The distribution of studies per country is depicted in Figure 3. The methodologies varied, with n = 38 (73%) being cross-sectional, n = 7 (13%) experimental longitudinal, n = 4 (8%) observational longitudinal, n = 2 (4%) randomized controlled trials, and n = 1 (2%) non-randomized controlled trials. Of the 52 studies, n = 7 (13%) exclusively involved women, n = 9 (17%) focused on children, n = 1 (2%) included both women and children, and n = 2 (4%) was focusing on men. Fourteen studies (27%) included participants diagnosed with asthma or COPD, whereas n = 38 studies (73%) focused on participants exposed to risk factors for obstructive lung diseases (Table 1).

**TABLE 1:** Overview of study characteristics of included studies (scoping review, low- and middle-income countries, 2022–2024).

Studies (n = 52, of which 6 multinational)				
Countries			Participants	
	n	%	n	%
Total	**61**	**100**	**244,004**	**100**
Sub-Saharan Africa	43	70.5	76,481	31.3
Nigeria	12	19.7	9,268	3.8
Uganda	10	16.4	19,905	8.2
Ghana	3	4.9	21,237	8.7
Malawi	3	4.9	2,256	0.9
Cameroon	2	3.3	4,646	1.9
Ethiopia	2	3.3	557	0.2
Tanzania	2	3.3	815	0.3
Kenya	2	3.3	2,398	1
Benin	1	1.6	13,589	5.6
Ivory Coast	1	1.6	104	<0.1
Namibia	1	1.6	107	<0.1
Sudan	1	1.6	5	<0.1
Democratic Republic of the Congo	1	1.6	247	0.1
Madagascar	1	1.6	661	0.3
Zimbabwe	1	1.6	686	0.3
Central America				
Honduras	1	1.6	137	<0.1
Asia	16	26.2	166,598	68.3
Kyrgyzstan	4	6.6	1,332	0.5
India	4	6.6	156,823	64.3
Vietnam	3	4.9	1,080	0.4
Nepal	2	3.3	3,620	1.4
Bangladesh	1	1.6	3,496	1.5
Myanmar	1	1.6	207	<0.1
Sri Lanka	1	1.6	40	<0.1
Middle East				
Syria	1	1.6	788	0.3

Study design			Participants	
Total	n	%	n	%
Cross-sectional	38	73	233,808	96
Longitudinal interventional	7	13	3,259	1.3
Longitudinal observational	4	8	6,699	2.7
Randomized controlled trial	2	4	198	<0.1
Non-randomized controlled trial	1	2	40	<0.1

### Risk Factors for COPD and Asthma

**FIGURE 4:**
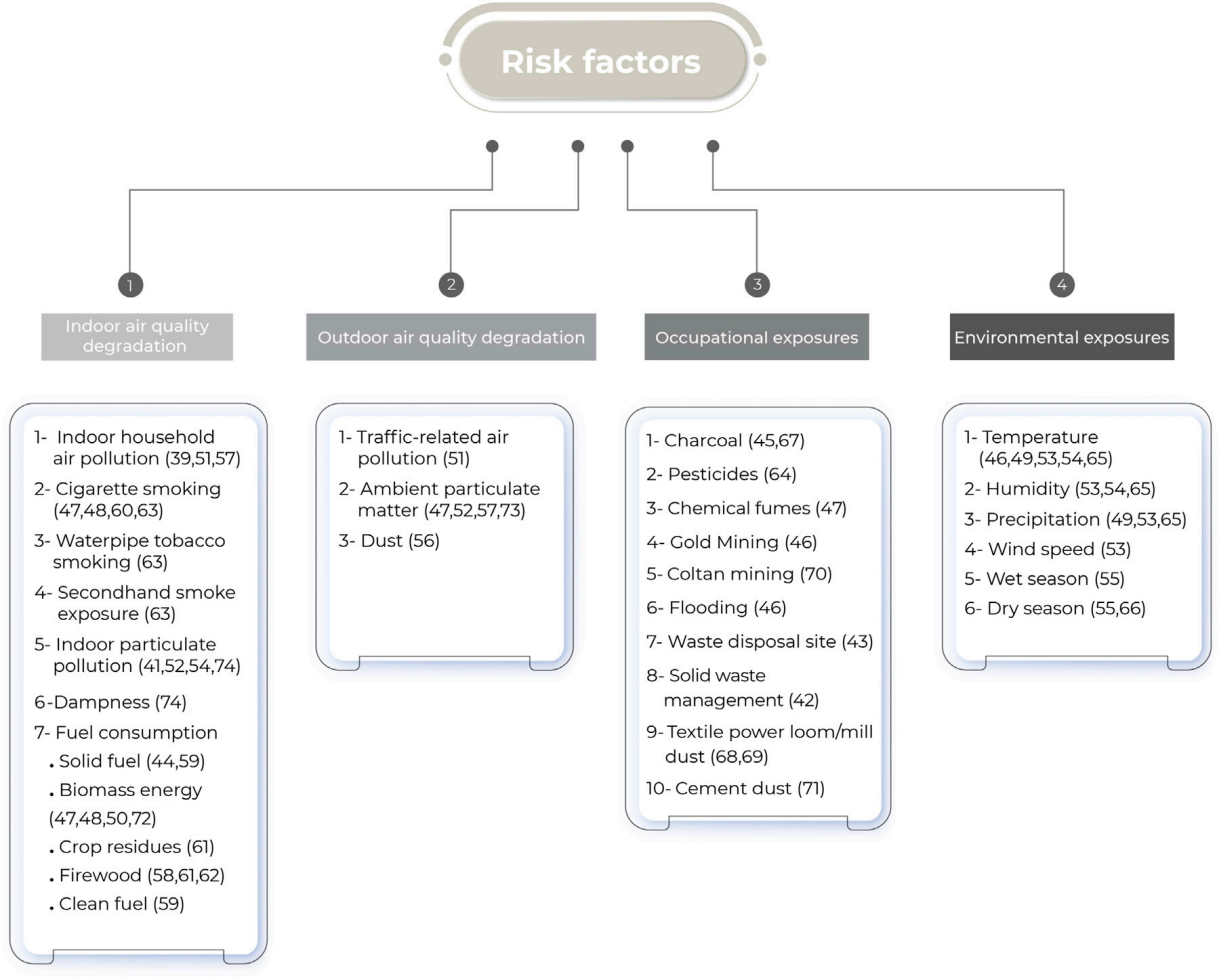
Categorization of risk factors identified in reviewed studies, grouped into four main domains: indoor air quality degradation, outdoor air quality degradation, occupational exposures, and environmental exposures (scoping review, low- and middle-income countries, 2022–2024).

Thirty-five studies (67%) [39, 41–74] reviewed the association between various risk factors and the incidence or severity of obstructive lung diseases, respiratory symptoms, and spirometry outcomes (see Figure 4). Disease status was determined using self-reports, hospital records, or spirometry results. Questionnaires were utilized to collect data on respiratory symptoms, and spirometry tests (see Supplementary Table S4) were used to measure lung function.

The questionnaires, designed to capture respiratory symptoms such as cough, sputum production, wheezing, and shortness of breath, were either developed by the researchers or adapted from established, validated instruments. These instruments include The Bold Core questionnaire, the Medical Research Council (MRC) questionnaire on respiratory symptoms, the Global Alliance Against Chronic Respiratory Diseases (GARD) World Health Organization (WHO) survey, the International Study of Asthma and Allergies in Childhood (ISAAC) questionnaire, and the European Community Respiratory Health Survey (ECRHS) questionnaire.

#### Indoor Air Quality Degradation

Eighteen (35%) studies [39, 41, 44, 47, 48, 50, 51, 52, 54, 57, 58, 59, 60, 61, 62, 63, 72, 74] explored the link between Indoor Air Quality degradation and lung diseases, respiratory symptoms, lung function tests, and disease severity (see Supplementary Table S5).

#### Outdoor Air Quality Degradation

Six (12%) studies [47, 51, 52, 56, 57, 73] assessed the relationship between outdoor air quality degradation and respiratory symptoms, as well as lung function outcomes (see Supplementary Table S6).

#### Occupational Exposures

Eleven (21%) studies [42, 43, 45, 46, 47, 64, 67, 68, 69, 70, 71] investigated the connection between occupational exposure and respiratory health outcomes (see Supplementary Table S7).

#### Environmental Exposures

Weather data was obtained through direct measurement, sourced from meteorological agencies, or inferred based on seasonal patterns (see Supplementary Table S8). Seven (13%) studies [46, 49, 53–55, 65, 66] investigated the relationship between environmental exposures and respiratory diseases (see Supplementary Table S9).

### Burden of Living With an Obstructive Lung Disease in Low-Resource Countries

Four (8%) studies [35, 40, 75, 76] estimated the personal and socioeconomic burden of living with an obstructive lung disease in low-resource settings. In a cross-sectional study conducted by Zoller et al. in Tanzania from 2015 to 2016, it was found that non-smokers refrained from taking a job due to shortness of breath. This condition was linked to high carboxyhemoglobin (SpCO) levels, living in rural areas, and the use of wood for cooking on open fires [75]. Brakema et al. found that asthma and COPD patients across Uganda, Vietnam, and Kyrgyzstan experienced low work absenteeism but significant activity impairment at work due to breathlessness, smoking, and solid fuel use [35]. In a qualitative study conducted by Tamire et al. in Addis Ababa, Ethiopia, it was found that COPD patients suffered from work impairment, decreased productivity, and limitations in physical activities due to their respiratory symptoms [35, 76]. These symptoms extended to financial, psychological, and social challenges, such as medication affordability issues, stress, helplessness, and reduced participation in social and religious events [76]. Similarly, Egere et al. explored challenges faced by chronic respiratory disease (CRD) patients - including asthma, COPD, occupational lung diseases, pulmonary hypertension, and cystic fibrosis - in Sudan and Tanzania. CRD resulted in reduced work capacity, economic hardship, and additional social and psychological burdens, including challenges in education expenses and caregiving for older relatives [40]. Healthcare-related costs, such as diagnostic tests and transportation, further burdened participants. Routine personal and community activities were severely impacted, leading to feelings of exclusion. Additionally, CRD patients faced stigmatization, often being wrongly associated with human immunodeficiency virus (HIV) or tuberculosis due to chronic cough, resulting in community isolation, limitations in sexual activities, and challenges in marital pursuits [40].

### Intervention Measures

Thirteen (25%) studies [36–38, 77–86] assessed various COPD and asthma intervention measures in low-resource settings, focusing on knowledge of the diseases and their risk factors, disease screening and control, treatment adherence, behavioral changes, and the use of improved indoor cookstoves (see Table 2).

**TABLE 2:** Intervention measures for chronic obstructive pulmonary disease and asthma in low-resource contexts based on included studies (scoping review, low- and middle-income countries, 2022–2024).

	Interventions	Outcomes	Study
Knowledge transmission	- COPD-specific education package [36]	- Increased knowledge about COPD: risk factors, management, prevention, etc.	[36]
	- A train-the-trainer lung health educational program [77]	- Improved knowledge about tobacco and biomass smoke	[77]
	- A train-the-trainer lung health educational program [78]	- Improved knowledge about tobacco and biomass smoke - Higher acceptability and adequate use of improved single and multiple pot stoves instead of open fire	[78]
	- Health education sessions on biomass smoke [80]	- Improved knowledge about tobacco and biomass smoke - Higher motivation for behavioral changes: reducing smoke emissions by using dry wood and improving kitchen ventilation	[80]
Disease and policy interventions	- COPD screening questionnaires in LMIC [38]	- Possible use of COPD screening questionnaires as a primary tool prior to spirometry	[38]
	- Health education sessions on biomass smoke [79]	- Modifications in cooking area, kitchen ventilation, and distance of children from smoke - Reduced expenditure on firewood and medical care related to smoke exposure	[79]
	- Enhanced asthma care package [81]	- Improved asthma management and adherence to medication - Decline in school absenteeism and emergency medical visits	[81]
	- Pharmacist-led asthma education [82]	- Improved asthma management and adherence to medication	[82]
	- Pulmonary rehabilitation sessions [86]	- Improved respiratory symptoms and exercise capacity	[86]
Improved cookstoves	- Implemented stoves using cleaner fuels and equipped with either chimneys, a combustion chamber, or a solar panel to charge the stove-fan battery [37, 83–85]	- High acceptance of the new cookstoves - Decreased self-reported respiratory symptoms - Decreased particulate matter concentration and air quality indoors	[37]
		- Unchanged self-reported respiratory symptoms - Improved spirometry test results	[83]
		- Decreased self-reported respiratory symptoms - Unchanged particulate matter concentration and air quality indoors - Improved spirometry test results - Reduced expenditure on additional fuel and decreased time spent collecting firewood	[84]
		- Increased self-reported respiratory symptoms - Decreased particulate matter concentration and air quality indoors - Unchanged spirometry test results	[85]

#### Knowledge Transmission

Four (8%) studies [36, 77, 78, 80] investigated educational interventions to enhance awareness of obstructive lung diseases and risk factors using tools like posters, flip charts, brochures, and local radio broadcasts (see Supplementary Table S10). In Uganda, a successful train-the-trainer program improved healthcare and community health workers’ understanding of tobacco and biomass smoke’s impact on lung health, resulting in increased questionnaire scores. This knowledge was effectively communicated to villagers, particularly regarding the dangers of tobacco smoke [77]. The program, adapted for Kyrgyzstan and Vietnam, led to a significant increase in correct responses on knowledge questionnaires, indicating sustained behavioral changes such as greater acceptance and proper use of lower-emission cookstoves [78]. Robertson et al. observed improved COPD knowledge in Nepal, Peru, and Uganda after distributing a COPD-specific educational package by community health workers [36]. Furthermore, educational sessions conducted by midwives in Uganda not only increased pregnant women’s knowledge about biomass smoke as an asthma risk factor by 45% but also motivated them to switch to safer fuel types and enhance kitchen ventilation [80].

#### Approaches to disease Management and Policy Interventions

Five (10%) studies [38, 79, 81, 82, 86] focused on interventions that improved COPD screening, asthma management, new respiratory health policy implementation and, rehabilitation and treatment of COPD in LMICs (see Supplementary Table S11).

##### COPD Screening

In these settings, three screening questionnaires - the COPD Assessment in Primary Care to Identify Undiagnosed Respiratory Disease and Exacerbation Risk (CAPTURE), the Lung Function Questionnaire (LFQ), and the COPD in LMICs Assessment (COLA-6) - were evaluated for their efficacy in identifying individuals diagnosed with COPD who would benefit from further spirometry testing [38]. The study found that participants diagnosed with COPD often had severe obstruction and were mostly unaware of their condition, despite experiencing a low quality of life. Those with false-positive results typically had a history of asthma or chronic bronchitis and were currently exposed to biomass smoke [38].

##### Asthma Management

In a randomized control trial (RCT), non-physicians delivered an enhanced asthma care package, including clinical assessment, optimized inhaled treatments, and customized asthma education, while the control group received standard physician care [81]. The intervention group showed fewer school absences, reduced emergency visits, and improved asthma control and medication adherence [81]. Another RCT assessed a pharmacist-led asthma education intervention [82], delivering information on triggers, control methods, and inhalers through clinic visits, mobile phone calls, and texts, compared to standard hospital care for the control group. The intervention resulted in improved asthma control and medication adherence [82].

##### Educational and Policy Interventions on Respiratory Health

Cartwright et al. evaluated the long-term effects of educational sessions on biomass smoke, initially investigated by Nantanda et al. [80], which were conducted by midwives for pregnant women and mothers [79]. The study observed that participants made modifications in their cooking areas, enhanced kitchen ventilation, and reduced children’s proximity to smoke while cooking [79]. Furthermore, a reduction in expenses for wood and medical care associated with smoke exposure was also noted [79].

##### Rehabilitation and Treatments in Low-Resource Settings

In Sri Lanka, a non-randomized controlled trial adapted the University Hospital of Leicester’s pulmonary rehabilitation protocol for COPD individuals in low-resource settings. The 6-week program included supervised and home-based exercises such as walking, stretching and strength training using simple equipment like water-filled bottles. Significant improvements were noted in respiratory symptoms, dyspnea, and exercise performance, with the intervention group surpassing the Minimum Clinically Important Difference (MCID) [86].

#### Low Emission Cookstoves

Four (8%) studies [37, 83–85] investigated the impact of introducing low-emission cookstoves indoors (see Supplementary Table S12). These stoves, utilizing cleaner fuels or enhancements like chimneys, combustion chambers and solar-powered stove-fan batteries led to reduced indoor particulate matter (PM2.5) [37, 85] and decreased self-reported respiratory symptoms [37, 84]. However, one study observed no change in cough or clinic visits after adoption [83]. Another study reported an increase in respiratory symptoms, attributed to altered reporting methods rather than a true increase [85]. Spirometry tests demonstrated improved lung function in Honduras and Kenya [83, 84], but no significant changes were noted in Malawi [85]. Improved cookstoves were well-received in rural communities across Uganda, Vietnam, and Kyrgyzstan [37], providing additional benefits such as reduced fuel costs and less time spent gathering firewood [84].

## Discussion

This review examines COPD and asthma in SSA and other LMICs, with a particular focus on the impact of climate change. It highlights key risk factors such as indoor and ambient air pollution, occupational hazards, and environmental allergens, impacting disease severity and exacerbations. Individuals with these conditions face multifaceted challenges including reduced work capacity, limited access to healthcare, social stigma, financial strain and psychological stress [87, 88]. Biomass fuel use for cooking exacerbates COPD in LMICs [87], particularly affecting women and children in rural areas [89]. Additionally, cultural practices such as burning incense, using kerosene lamps, and lighting mosquito coils contribute to higher levels of indoor pollution [89]. Interventions such as educational programs, non-physician-led treatments, and improved cookstove initiatives aim to mitigate indoor air pollution and improve respiratory outcomes. Aging also appears to significantly contribute to the prevalence of COPD in SSA. Between 1990 and 2019, the overall prevalence of COPD cases in SSA increased by 117%. However, during the same period, the age-standardized COPD prevalence rate declined by 3.3% [7]. The decline in the age-standardized COPD prevalence rate in SSA, amidst an overall case increase, might result from improved healthcare, public health policies, and a younger population.

Greenhouse gas concentrations have increased rapidly since 1850, in parallel with a 1.1°C increase in global surface temperature by 2011–2020 compared to 1850–1900 [90]. The regions that have generally contributed the least to CO2 emissions and climate change are considered to be the most vulnerable [91] and are mainly located in East, Central and West Africa, with some other regions in South Asia and Central and South America [90]. Climate change can have direct and indirect effects on health; for example, exposure to extreme heat can have indirect effects through increased transmission of food- and waterborne diseases, and direct effects through dehydration, heat stroke, and increased hospitalization and mortality from respiratory and cardiovascular diseases [92].

Although no study in the literature has explicitly examined the relationship between climate change and chronic respiratory diseases in SSA and other LMICs, it can be argued that some of the risk factors exacerbating COPD and asthma developed in this review may serve as mediators between climate change and chronic respiratory diseases. Thus, a hypothesized link between both entities in SSA and other LMICs can be deduced.

Weather variables like temperature, humidity, and precipitation play a significant role in obstructive lung diseases. Studies exhibit inconsistent outcomes, potentially attributable to climate variations or differing methodologies and settings; for example, a study conducted in Sudan’s dry Savanna region revealed an inverse correlation between temperature and asthma severity [53]. This contrasts with the common understanding that high temperatures worsen respiratory conditions [93]. The wet season with low temperatures and high humidity has been associated with increased asthma cases owing to a greater prevalence of viral infections and indoor triggers [54], which is consistent with previous research, highlighting the role of humidity and the presence of allergens and pollutants in indoor environments [94]. The combination of low temperature and humidity can cause bronchoconstriction, underscoring the importance of maintaining suitable indoor climates [95]. High humidity can exacerbate asthma by releasing allergens, highlighting the importance of indoor air quality management [95]. Although wind can increase long-range transport of pollen and pollutants [96], increased outdoor wind speed can help disperse pollutants, potentially providing protective benefits that require further investigation [95]. Human-induced climate change associated with increased greenhouse gas emissions affects weather variables, leading to heat waves, heavy precipitation and droughts in some regions [90]. Climate change may therefore have direct and indirect effects on respiratory health [97]. The included studies in this review however focused on variations of climate factors rather than extreme weather events caused by climate change. Further investigation should therefore be implemented, particularly in highly affected areas such as SSA, to study the complex relationship between climate change and chronic respiratory diseases.

In this review, other risk factors listed in Figure 4 have been shown to be associated with reduced lung function.

Ambient air pollution, divided into gaseous and particulate matter (PM) pollutants, affects both indoor and outdoor air quality. Gaseous pollutants include nitrogen dioxide (NO2), sulfur dioxide (SO2), ozone (O3), carbon monoxide (CO), carbon dioxide (CO2), and volatile organic compounds (VOCs). PM pollutants, on the other hand, are classified by size and are formed from gases such as nitrogen oxides, ammonia, SO2, and VOCs [97]. In this review, studies have shown that indoor biomass and coal burning for cooking and heating in SSA and other LMICs has led to an increase in indoor gaseous and PM pollutants, and therefore to a degradation of indoor air quality. Exposure to dust and traffic-related air pollution has led to an increase in outdoor PM pollutants, resulting in poor outdoor air quality. PM pollutants have been shown to vary seasonally, with higher levels in winter due to increased dust particles and biomass burning [97]. Specifically, in SSA, excessive heat and heavy rainfall during the rainy season make outdoor cooking impractical, leading people to burn biomass indoors more frequently, resulting in poorer indoor air quality. In addition, future intense heat waves caused by climate change will lead to more wildfires, which will increase particulate matter and harmful greenhouse gases and lead to deterioration of outdoor air quality [98]. Dry and hot conditions and reduced precipitation can also lead to increased air pollution, especially ozone [97, 98], due to increased photochemical production [98]. Climate change and air pollution have also been shown to be interrelated, as PM pollutants and greenhouse gases are often co-emitted [99]; burning fossil fuels not only increases air pollution but also produces greenhouse gases, which in turn lead to extreme weather events such as droughts, heat waves and floods, which can further affect respiratory health [97]. Charcoal production and climate change are also linked through a feedback loop rather than a direct cause and effect relationship. The process of charcoal production involves deforestation and the release of greenhouse gases such as CO2 and methane, which in turn contribute to global warming [100]. In some regions affected by climate change, particularly drought, farmers may be more likely to engage in charcoal production during the dry season. In Tanzania, farmers rely on charcoal production as an alternative source of income after crop failure during the dry season [101]. This cyclical relationship will lead to poorer respiratory health. Pesticide use and climate change have also been shown to be interconnected. Rising temperatures allow for severe pest outbreaks because such conditions allow pests to survive in regions where they were previously unable to thrive [102]. In addition, the lengthening of the growing season due to higher temperatures exposes plants to pests for longer periods of time [103]. This leads to increased use of pesticides to control pest infestations. Pesticides, in turn, contribute to CO2 emissions and can disrupt soil health by killing microorganisms that play a beneficial role in carbon sequestration [104], resulting in the release of more CO2 into the atmosphere and exacerbating climate change.

Implementing cookstoves that use cleaner fuels and are equipped with a solar panel to charge the stove-fan battery [85] is an effective way that allows individuals to cook indoors during extreme heat and heavy rainfall while maintaining a good indoor air quality and generating less CO2 emissions. On the other hand, cookstoves with chimneys or a combustion chamber may reduce indoor air pollution but will still contribute to greenhouse gas emissions [83, 84]. In addition, the introduction of educational programs to increase knowledge about biomass smoke [77, 78, 80] can play a key role in reducing the burning of indoor biomass fuel but does not offer an established long-term alternative that can be afforded by the general population. These solutions can then be considered as important intermediate steps in LMICs, where resources are scarce and the use of clean energy and electric stoves is not always feasible.

Sustainable agricultural practices and predominantly plant-based diets decrease emission of greenhouse gases [98]. Adopting diets rich in antioxidants and vitamins, characteristic of Mediterranean or “prudent” diets, can significantly improve pulmonary function and reduce asthma and COPD risks [105–108]. However, over two million people suffer micronutrient deficiencies [109], especially in SSA [110]. Educational initiatives in LMICs promoting healthy diets typically utilize printed and digital materials, often based on behavior change models, and aim to engage both women and men, reflecting their distinct roles in food preparation and decision-making [111–113]. Climate change is likely to exacerbate nutrient deficiency in LMICs by affecting food security, namely accessibility, availability and food stability [114, 115]. However, other key factors contribute to food insecurity in LMICs, including limited access to modern agricultural technologies, reliance on subsistence farming, and political and economic instability in conflict zones, which disrupt agricultural production and reduce access to food [116].

It is evident that climate change plays a pivotal role in the prevalence of chronic respiratory diseases in SSA and other LMICs. This impact can be achieved directly or indirectly through various mediators, including indoor and outdoor air pollution, charcoal production, pesticide use, and nutrient deficiency. However, additional factors unique to SSA contribute to the heightened burden of chronic respiratory diseases and impede their prevention.

In certain regions in SSA where targeted asthma management protocols are lacking, managing risk factors and promoting lifestyle changes are prioritized [117]. The shortage of trained medical professionals, particularly in remote areas, poses a challenge for diagnosing and managing obstructive lung diseases. Community-based screening, technology utilization, and awareness initiatives are crucial for enhancing primary healthcare [89]. Spirometry plays a vital role in diagnosing obstructive lung diseases, but inconsistencies in diagnosis can arise from varying cutoff measures [7]. In countries like South Africa and Nigeria, spirometry use is largely confined to specialized hospitals and research settings, despite being recommended for asthma diagnosis. In Tanzania, asthma diagnosis relies mainly on clinical history and findings, while Zimbabwe lacks specific asthma diagnostic guidelines [117]. Challenges in asthma diagnosis encompass not only spirometry availability and clinician training but also equipment maintenance, electricity access, technical support, and the use of outdated equipment, necessitating comprehensive training and guidelines for healthcare workers [89, 118]. Implementing tailored pulmonary rehabilitation programs and national strategies can alleviate the burden of lung diseases in low-resource settings [89, 118, 119], although challenges like infrastructure limitations, budget constraints, political instability and healthcare workforce shortages persist in implementing WHO’s NCD management framework [11, 120].

Enhanced research, screening, and interventions are crucial for obstructive respiratory diseases in LMICs, especially in SSA, where the need is intensified by the disproportionate impact of climate change-induced global warming [119].

### Limitations

In our review, we focused exclusively on studies from LMIC contexts, a deliberate choice that, while limiting comparative insights from high-resource settings, was aimed at a specific analysis. The included studies varied in their definitions of certain risk factors and were influenced by multiple confounders, necessitating cautious interpretation of direct comparisons. Additionally, we omitted studies that described but did not empirically test intervention measures, which could have offered a wider view on potential intervention strategies despite their lack of practical outcome data.

### Conclusion

This review examines the impact of different risk factors on the health of individuals with COPD and asthma in LMICs. Climate change plays an indirect but pivotal role in the exacerbation of the burden of chronic respiratory diseases by impacting some risk factors specific to SSA and other LMICs.

This research highlights the need for comprehensive strategies that encompass educational programs, low-emission cookstoves and the implementation of targeted healthcare policy to mitigate the effects of climate change on respiratory health. Furthermore, it emphasizes the urgent necessity for further interventions that are tailored to the specific environmental and socio-economic context. In order to address these challenges, it is essential that there is urgent global collaboration and investment in research and infrastructure with the aim of improving public health resilience against climate-related exacerbations of respiratory conditions in LMICs.

## Abbreviations

CAPTURE, COPD Assessment in Primary Care to Identify Undiagnosed Respiratory Disease and Exacerbation Risk; COLA-6, COPD in LMICs Assessment; COPD, Chronic obstructive pulmonary disease; CRD, Chronic Respiratory Disease; ECRHS, European Community Respiratory Health Survey; FEV1, Forced Expiratory Volume in 1 s; FVC, Forced Vital Capacity; GARD, Global Alliance Against Chronic Respiratory Diseases; HIV, Human Immunodeficiency Virus; ISAAC, International Study of Asthma and Allergies in Childhood; LAMAs, Long-acting muscarinic antagonists; LFQ, Lung Function Questionnaire; LMICs, Low-and middle-income countries; MCID, Minimum Clinically Important Difference; MRC, Medical Research Council; NCD, Non-communicable disease; PEFR, Peak Expiratory Flow Rate; PEO, Population-Exposure-Outcome; PM, Particulate Matter; PRISMA-ScR, Preferred Reporting Items for Systematic Reviews and Meta-Analyses extension for Scoping Reviews; RCT, Randomized Control Trial; SpCO, Carboxyhemoglobin saturation; SSA, Sub-Saharan Africa; WHO, World Health Organization; WMO, World Meteorological Organization.
