# Supplementary material for: Understanding the Risk Factors, Burden, and Interventions for Chronic Respiratory Diseases in Low- and Middle-Income Countries: A Scoping Review
Source: Public Health Rev. 2024 Oct 31;45:1607339. doi: 10.3389/phrs.2024.1607339 (PMC11560431; doi:10.3389/phrs.2024.1607339)
Supplement: Supplementary file 1 [file DataSheet1.docx]

**Supplementary material**

**Table 1.** Inclusion and exclusion criteria based on the population-exposure-outcome framework.

|  | Inclusion | Exclusion |
| --- | --- | --- |
| Population | - Study participants living in LMICs | - Study participants living in high-income countries |
| Exposure | - Manifestations and symptoms associated with COPD and asthma - Risk factors leading to COPD and asthma | - Studies not focusing on COPD and asthma or their risk factors |
| Outcome | - Studies examining exposures to risk factors for COPD and asthma, including indoor air pollution, heat stress, smoking, dust, and other environmental hazards - Studies assessing preventive and management strategies for COPD and asthma within LMICs - Exploration of functional impairments linked to COPD and asthma in LMICs | - Studies detailing only the prevalence of COPD/asthma and associated risk factors - Studies highlighting the lack of appropriate intervention measures - Studies evaluating perceptions related to COPD and asthma - Studies presenting interventions without empirical validation |
| Time | - Studies published after January 1^st^, 2011 | - Studies published before January 1^st^, 2011 |
| Study type | - Full text available - Any primary research | - Only abstract available - Secondary/synthesis research |
| Language | - English | - Non-English |

**Table 2.** Search strings (last search on 04.01.2024)

| Database | Search strings |
| --- | --- |
| PubMed | (asthma OR ("asthma"[MeSH Terms]) OR ("asthma chronic obstructive pulmonary disease overlap syndrome"[MeSH Terms]) OR "COPD" OR "chronic obstructive lung disease" OR "chronic obstructive pulmonary disease") AND (("smoking"[MeSH Terms]) OR "household air pollution" OR "indoor air pollution" OR "biomass fuel" OR "fume" OR "dust exposure" OR "high temperature" OR ("climate change"[MeSH Terms]) OR "pesticide" OR ("greenhouse effect"[MeSH Terms]) OR ("global warming"[MeSH Terms]) OR drought* OR "heat exposure" OR ("hot temperature"[MeSH Terms]) OR "weather extreme*" OR "extreme weather event*" OR ("climate"[MeSH Terms]) OR "hot weather" OR "hot weather condition*" OR "extreme climate" OR "extreme climate change" OR ("extreme environments"[MeSH Terms]) OR "heat wave" OR "hot environment") AND (("africa south of the sahara"[MeSH Terms]) OR "sub saharan Africa" OR "low resource setting*") AND (recommendation* OR management* OR "pulmonary rehabilitation" OR "education program" OR ("education"[MeSH Terms]) OR "disease exacerbation" OR exacerbation* OR "work capacity" OR "physical capacity") |
| Embase | ('chronic obstructive lung disease' OR 'asthma' OR 'asthma-chronic obstructive pulmonary disease overlap syndrome') AND ('smoking' OR 'household air pollution' OR 'indoor air pollution' OR 'biomass fuel' OR 'fume' OR 'dust exposure' OR 'heat stress' OR 'high temperature' OR 'climate change' OR 'pesticide' OR 'greenhouse effect' OR 'global warming potential' OR 'drought' OR 'thermal exposure' OR 'extreme weather' OR 'climate' OR 'hot weather' OR 'extreme environment' OR 'heat wave' OR 'hot environment') AND ('Africa south of the Sahara' OR 'sub saharan african' OR 'low resource setting') AND ('recommendations' OR 'management' OR 'pulmonary rehabilitation' OR 'education' OR 'disease exacerbation' OR 'work capacity' OR 'physical capacity') |
| Scopus | ("chronic obstructive lung disease" OR COPD OR "chronic obstructive pulmonary disease" OR asthma OR "asthma-chronic obstructive pulmonary disease overlap syndrome") AND (smoking OR "household air pollution" OR "indoor air pollution" OR "biomass fuel" OR fume OR "dust exposure" OR "heat stress" OR "high temperature" OR "climate change" OR pesticide OR "greenhouse effect" OR "global warming" OR drought OR "heat exposure" OR "hot temperature" OR "weather extreme" OR "extreme weather event" OR climate OR "hot weather" OR "hot weather condition" OR "extreme climate" OR "extreme climate change" OR "extreme environments" OR "heat wave" OR "hot environment") AND ("sub saharan africa" OR "low resource setting" OR "Africa south of the Sahara") AND (recommendations OR management OR "pulmonary rehabilitation" OR education OR "disease exacerbation" OR "work capacity" OR "physical capacity") |
| Google scholar | (COPD OR asthma) AND ("climate change" OR "indoor air pollution" OR "high temperature" OR "heat stress" OR fumes) AND ("sub-saharan Africa" OR "low-resource setting") AND (recommendations OR management OR "pulmonary rehabilitation" OR "work capacity" OR education) |

**Table 3.** Demographics of included studies.

| Author (study) | Country | Year | Study population | Total number of study participants | Sex (male)% | Sex (female)% | Age of participants (years) | Outcome |
| --- | --- | --- | --- | --- | --- | --- | --- | --- |
| Eghomwanre and Oguntoke (1) | Nigeria | 2022 | Asthmatic children | 304 | 51.6 | 48.4 | Range 0-17 | Assessment of the relation between various risk factors and the occurrence of obstructive lung disease and/or the severity of the disease, respiratory symptoms, and lung function tests |
| Munyai and Nunu (2) | Zimbabwe | 2020 | General population living around waste collection points | 686 | 42.1 | 57.9 | Range 26-35 |  |
| Akpeimeh et al. (3) | Nigeria | 2019 | Workers at dumpsite | 149 | 87 | 13 | Median 30 |  |
| Obaseki et al. (4) | Nigeria | 2017 | General population | 1,147 | 37 | 63 | 40+ |  |
| Obiebi et al. (5) | Nigeria | 2017 | Charcoal and non-charcoal workers | 296 | 37 | 63 | 39.14 (SD 15.22) for charcoal group  38.27 (SD 14.78) for control group |  |
| Ayaaba et al. (6) | Ghana | 2017 | Miners | 1,001 | 100 | 0 | Range 20-73 (median 35) |  |
| Hamatui and Beynon (7) | Namibia | 2017 | General population | 107 | 51 | 49 | ≥ 18 |  |
| Agrawal and Sutapa (8) | India | 2012 | General population | 156,316 | 36.3 | 63.7 | Range 20-49 |  |
| Désiré et al. (9) | Cameroon | 2015 | Asthma patients | 4,346 | 43 | 57 | All ages |  |
| Pathak et al. (10) | India | 2018 | Females from general population | 310 | 0 | 100 | 42.52 (SD 10.22): biomass group  37.36 (SD 10.78): LPG group |  |
| Mustapha et al. (11) | Nigeria | 2011 | Children from general population | 1,397 | 48 | 52 | Range 7-14 |  |
| René et al. (12) | Ivory Coast | 2019 | Children from general population | 104 | 52 | 48 | <5 |  |
| Abbas et al. (13) | Nigeria | 2021 | Asthma patients | 87 | 31 | 69 | 32.1 (SD 10.8) |  |
| Eghomwanre et al. (14) | Nigeria | 2022 | Asthmatic children | 4,126 | - | - | ≤ 17 |  |
| Desalu, O. O. (15) | Nigeria | 2011 | Asthma and COPD patients | 99 | - | - | >15 |  |
| McElroy et al. (16) | Benin | 2022 | Children from general population | 13,589 | - | - | <5 |  |
| Oyebanji et al. (17) | Nigeria | 2021 | Farmers and non-farmers | 390 | 67 | 33 | All ages |  |
| Woolley et al. (18) | Uganda | 2020 | Children from general population | 12,161 | 49.8 | 50.2 | Under 5 |  |
| Tamire et al. (19) | Ethiopia | 2020 | Females from general population | 545 | 0 | 100 | 30.3 (SD 6.1) |  |
| Brakema et al. (20) | Kyrgyzstan | 2019 | General population | 392 | 47.7 | 52.3 | 44.4 (SD 13.6) in lowlanders  50.0 (SD 16.3) in highlanders |  |
| Siddharthan et al. (21) | Uganda, Peru, Chile, Argentina, Uruguay and Bangladesh | 2018 | General population | 12,396 | 51.5 | 48.5 | 54.9 (SD 10.9)  Range 35-95 |  |
| Das et al. (22) | Malawi | 2017 | Primary cooks in rural vs urban households | 655 | - | - | 37.3 (SD 13.8)  Range 17-72 |  |
| Ngahane et al. (23) | Cameroon | 2015 | Females from general population | 300 | 0 | 100 | 40+ |  |
| Mohammad et al. (24) | Syria | 2013 | Females from general population | 788 | 0 | 100 | ≥ 6 |  |
| Quansah et al. (25) | Ghana | 2019 | Farmers and non-farmers | 310 | 74.5 | 25.5 | 44 (SD 7.5) |  |
| Omonijo et al. (26) | Nigeria | 2011 | Patients with respiratory diseases | 599 | 53 | 47 | All ages |  |
| Narh et al. (27) | Ghana | 2021 | Asthmatic patients | 19,926 | 39.8 | 60.2 | 33.7 (SD 24.6) |  |
| O. O. Olujimi (28) | Nigeria | 2016 | Charcoal and non-charcoal workers | 596 | 72.5 | 27.5 | 32.67 (SD 10.47): charcoal group  35.46 (SD 12.82): control group |  |
| Subramaniam et al. (29) | India | 2023 | Textile power loom workers | 97 | 75.25 | 24.74 | 45 (SD 11.2) |  |
| Oo et al. (30) | Myanmar | 2021 | Textile mill workers | 207 | 9.2 | 90.8 | 38.8 (SD 11.2) |  |
| Leon-Kabamba et al. (31) | Congo | 2018 | Coltan miners | 247 | - | - | 32.79 (SD 8.34) |  |
| Dushyant et al. (32) | India | 2023 | Cement factory workers | 100 | 100 | 0 | 40.1 (SD 11.4) |  |
| Jestin-Guyon et al. (33) | Madagascar | 2023 | Children under 15 years old | 661 | - | - | 6.7 (SD 4.6) |  |
| Meme et al. (34) | Kenya | 2023 | Schoolchildren | 2,373 | 47.8 | 52.2 | Range 4-18 |  |
| Wafula et al. (35) | Uganda | 2023 | General population of an informal settlement | Adults: 284 Children: 187 | Adults: 14.8 Children: 47.4 | Adults: 85.2 Children: 52.6 | Median: Adults: 29 Children: 2 |  |
| Zoller et al. (36) | Tanzania | 2022 | COPD patients | 808 | 49 | 51 | Median:46 | Assessment of the burden of living with an obstructive lung disease |
| Tamire et al. (37) | Ethiopia | 2021 | General population | 12 | 58 | 42 | Range 45-78 |  |
| Brakema et al. (38) | Uganda, Vietnam, Kyrgyzstan and Greece | 2019 | COPD and/or asthma patients | 1,040 | 53.3 | 46.7 | Median: 60 |  |
| Egere et al. (39) | Sudan and Tanzania | 2023 | Chronic respiratory diseases | 12 | 42 | 58 | >15 |  |
| Robertson et al. (40) | Nepal, Peru and Uganda | 2021 | COPD patients | 196 | 58.7 | 41.3 | 66 (SD 11.1) | Intervention measures that enhance knowledge about obstructive lung diseases |
| Jones et al. (41) | Uganda | 2019 | General population | 1,244 | 53 | 47 | 37 (SD 15.7) |  |
| Brakema et al. (42) | Kyrgyzstan and Vietnam | 2020 | General population | 920 | - | - | - |  |
| Nantanda et al. (43) | Uganda | 2019 | Pregnant and post-natal females | 88 | 0 | 100 | - |  |
| Siddharthan et al. (44) | Nepal, Peru and Uganda | 2022 | General population | 10,709 | 50 | 50 | 56.3 (SD 11.7) | Intervention measures that enhance screening, disease control and adherence to treatment and/or behavioral changes |
| Cartwright et al. (45) | Uganda | 2022 | Pregnant and post-natal females | 12 | 0 | 100 | - |  |
| Rylance et al. (46) | Malawi | 2021 | Asthmatic children | 120 | 65.8 | 34.2 | 9.8 (SD 2.8) |  |
| Amorha et al. (47) | Nigeria | 2020 | Asthmatic patients | 78 | 38.5 | 61.5 | ≥ 16 |  |
| Sooriyakanthan et al. (48) | Sri Lanka | 2022 | COPD patients | 40 | 100 | 0 | Intervention: 67.1 ± 6.8  Control: 68.5 ± 6.5 |  |
| Van Gemert et al. (49) | Uganda, Vietnam and Kyrgyzstan | 2019 | General population | 649 | 33 to 50 | 50 to 67 | Adult age range: 36.2 to 52.1  Children age range: 2.8 to 8.7 | Implementation of low emission cookstoves indoors |
| Rennert et al. (50) | Honduras | 2015 | Children and females from general population | 137 | 32 | 68 | Females: ≥15  Children: ≥6 |  |
| Critchley et al. (51) | Kenya | 2015 | Females from general population | 25 | 0 | 100 | Range 25-50 |  |
| Rylance et al. (52) | Malawi | 2020 | General population | 1,481 | 43 | 57 | 43.8 (SD 17.8) |  |

**Table 4.** Characteristics of spirometry tests.

| Author (study) | Spirometer device | Measured parameters for assessment or diagnosis | Test administrators | Frequency of test administration |
| --- | --- | --- | --- | --- |
| Siddhartan et al. (21) | EasyOne (CRONICAS study) | COPD:  post-bronchodilator FEV1/FVC < 70% | Trained technicians | 3 (At 0, 20 and 40 months) |
|  | EasyOne (PRISA study) | COPD:  post-bronchodilator FEV1/FVC < 70%, post-bronchodilator FEV1/FVC < LLN and post-bronchodilator < 88% of the predicted value for men and < 89% for women | Trained personnel | 2 (At 0 and 4 years) |
|  | EasyOne (icddr,b study in Bangladesh) | COPD:  post-bronchodilator FEV1/FVC < 70% and post-bronchodilator FEV1/FVC < LLN | Field research assistants trained for 2 weeks on how to use the spirometer in the clinic and within the community | 1 |
|  | Pneumotrac with Spirotrac V software (FRESH AIR study) | COPD:  post-bronchodilator FEV1/FVC < LLN | Local nurses and health officers trained for 5 days on how to use the spirometer | 1 |
|  | EasyOne (LINK study) | - | - | - |
| Obaseki et al. (4) | EasyOne | Airway obstruction:  postbronchodilator FEV1/FVC ratio < LLN | Trained technicians | 1 |
| Ngahane et al. (23) | Spirobank II | Airway obstruction:  post bronchodilator FEV1/FVC < 70% | Trained assistant and results reviewed by a pulmonologist | 1 |
| Brakema et al. (20) | EasyOne | COPD:  post-bronchodilator FEV1/FVC ratio < 70% | Medical doctors trained on how to use the spirometer | 1 |
| Tamire et al. (19) | EasyOne | Airway obstruction:   1. Pre-bronchodilator FEV1/FVC ratio <70% and FEV1<80% of predicted 2. Pre-bronchodilator FEV1/FVC ratio <LLN   Asthma: FEV1 > 200mL and FEV1 > 12% reversibility after bronchodilator administration | Trained and experienced graduate nurses and health officers | 1 |
| Oyebanji et al. (17) | PiKo-1 | FEV1 and PEFR | Principal investigator | 1 |
| Obiebi et al. (5) | Micro-GP | Obstructive and restrictive lung diseases:  FEV1, FVC, FEV1/FVC and PEFR | Medical doctors trained on how to use the spirometer | 1 |
| O. O. Olujimi (28) | Piko-1 | FEV1 and PEFR | - | 1 |
| Mohammad et al. (24) | Spirobank II | Airway obstruction: pre-bronchodilator FEV1/FVC ratio <70% and FEV1<80% of predicted.   COPD:  post-bronchodilator FEV1/FVC ratio <70%  Asthma:  FEV1 > 200mL and FEV1 > 12% reversibility after bronchodilator administration | General practitioners trained on how to use the spirometer | 1 |
| Subramaniam et al. (29) | PC-based spirometer (Medical International Research, Italy) | Obstructive and restrictive lung diseases: FEV1, FVC, FEV1/FVC, PEFR, and FEF25–75 | - | 1 |
| Oo et al. (30) | Vitalograph In2itive model 2120, version 1.05 | Obstructive and restrictive lung diseases: FEV1, FVC and FEV1/FVC | Occupational hygienist | 1 |
| Leon-Kabamba et al. (31) | Wright Mini-Peak Flow meter (Airmed, Clement Clarke International, London) | PEFR | Medical doctors | 1 |
| Dushyant et al. (32) | EasyOne | FEV1, FVC, FEV1/FVC and PEFR | - | 1 |
| Meme et al. (34) | EasyOne | FEV1, FVC and FEV1/FVC | Trained technicians | 1 |

FEV1: Forced expiratory volume in 1 second, FVC: Forced vital capacity, LLN: Lower limit of normal, PEFR: Peak expiratory flow rate, FEF25–75: Forced expiratory flow, Bronchodilator: inhaled salbutamol or albuterol

**Table 5.** Summary of study findings on the relationship between indoor air quality degradation and obstructive lung diseases: impact on respiratory symptoms, lung function tests, and disease severity.

| Indoor air quality degradation | Lung Diseases | | | | Respiratory symptoms | | | | | | | Lung function tests | | | Severity |
| --- | --- | --- | --- | --- | --- | --- | --- | --- | --- | --- | --- | --- | --- | --- | --- |
|  | COPD | Asthma | Chronic bronchitis | Emphysema | Dyspnea | Dyspnea at rest | Dyspnea on exertion | Cough | Wheeze | Phlegm | Not specified | FEV1 | FVC | FEV1/FVC | COPD |
| Indoor household air pollution | (+): (21)*,(20) |  |  |  |  |  |  | (∅): (11) | (∅): (11) | (∅): (11) | (+): (21) | (-): (21) | (-): (21) | (-): (21) | (+): (21) |
| Cigarette smoking | (+): (20,24) | (+): (8)*, (7)  (∅): (24) |  |  |  |  |  | (+): (24) | (∅): (24) | (+): (24) |  |  |  |  |  |
| Waterpipe tobacco smoking | (∅): (24) |  |  |  |  |  |  |  | (+): (24) | (∅): (24) |  |  |  |  |  |
| Secondhand smoke exposure | (+): (24) | (∅): (24) |  |  |  |  |  |  |  |  |  |  |  |  |  |
| Indoor particulate pollution |  | (∅): (1)* (+): (14) |  |  |  |  |  | (+): (35) | (+): (12) |  |  |  |  |  |  |
| Dampness |  |  |  |  | (+): (35) |  |  | (+): (35)* |  | (+): (35), (35)* |  |  |  |  |  |
| Solid fuel |  |  |  |  |  |  |  | (+): (4)*, (19) |  | (+): (4)*, (19) |  | (-): (19) | (∅): (19) | (∅): (4,19) |  |
| Biomass energy |  | (+): (8)*,(10) |  |  |  |  |  | (+): (7) |  |  | (+): (33) |  |  |  |  |
| Crop residues |  |  |  |  |  | (+): (22) |  |  |  | (+): (22) |  |  |  |  |  |
| High-quality firewood |  |  |  |  |  |  |  |  |  | (-): (22) |  |  |  |  |  |
| Low-quality firewood |  |  |  |  |  |  | (+): (22) |  |  | (+): (22) |  |  |  |  |  |
| Firewood |  |  | (+): (23) |  | (+): (18) |  |  | (+): (18) |  |  |  | (-): (23) |  |  |  |
| Clean fuel |  |  |  |  |  |  |  |  |  |  |  |  | (∅): (19) | (∅): (19) |  |

(21)*: stronger in women and older age (≥55 years old), (8)*: in both women and men, (1)*: in dry and wet season*,* (4)*: stronger in women, (35): in adults, (35)*: in children

**Table 6.** Summary of study findings on the associations between outdoor air quality degradation, respiratory symptoms, and lung function test outcomes.

| Outdoor air quality degradation | Respiratory symptoms | | | | Lung function tests | | | |
| --- | --- | --- | --- | --- | --- | --- | --- | --- |
|  | Cough | Wheeze | Phlegm | Dyspnea | FEV1 | FVC | FEV1/FVC | PEFR |
| TRAP | (+): (11) | (+): (11) | (+): (11) |  |  |  |  |  |
| Ambient particulate matter | (+): (7,12) | (+): (12,34) | (+): (7) | (+): (34) | (-): (17) |  |  | (-): (17) |
| Dust | (+): (16) |  |  |  |  |  |  |  |

**Table 7.** Summary of study findings on the associations between occupational exposures and obstructive lung diseases, respiratory symptoms, and lung function tests.

| Occupational exposures | Diseases | | | | Respiratory symptoms | | | | | | Lung function tests | | | | |
| --- | --- | --- | --- | --- | --- | --- | --- | --- | --- | --- | --- | --- | --- | --- | --- |
|  | Asthma | Chronic bronchitis | Emphysema | Obstructive lung | Dyspnea | Cough | Wheeze | Phlegm | Asthma symptoms | Not specified | FEV1 | FVC | FEV1/FVC | PEFR | FeNO |
| Charcoal |  |  |  | (∅): (5) |  |  |  |  |  |  | (+): (5) (-): (28) | (+): (5) |  | (∅): (5) (-): (28) |  |
| Pesticides |  |  |  |  | (∅): (25) | (+): (25) | (+): (25) |  |  |  |  |  |  |  |  |
| Chemical fumes |  |  |  |  |  | (-): (7) |  | (-): (7) |  |  |  |  |  |  |  |
| Gold mining |  |  | (+): (6) |  |  |  |  |  |  |  |  |  |  |  |  |
| Coltan mining |  |  |  |  | (+): (31) | (+): (31) | (+): (31) | (+): (31) |  |  |  |  |  | (-): (31) |  |
| Flooding | (+): (6) | (+): (6) |  |  |  |  |  |  |  |  |  |  |  |  |  |
| Waste disposal sites | (∅): (3) |  |  |  |  | (∅): (3) |  | (∅): (3) |  |  |  |  |  |  |  |
| Solid waste management | (+): (2) |  |  |  | (+): (2) | (+): (2) |  |  |  |  |  |  |  |  |  |
| Textile power loom/ mill dust |  |  |  |  |  |  |  |  |  | (+): (29) | (-): (29,30) | (-): (29,30) |  |  |  |
| Cement dust |  |  |  |  |  |  |  |  |  | (+): (32) |  |  | (-): (32) | (-): (32) |  |

**Table 8.** Characteristics of weather data.

| Author (study) | Study aspect | Country | Year | Source of weather data | Weather parameters | Duration | Frequency of measurement |
| --- | --- | --- | --- | --- | --- | --- | --- |
| Eghomwanre et al. (14) | Prospective | Nigeria | 2022 | Lutron 4 in 1 environmental tester (LM-8000) | Indoor temperature and relative humidity | 1 year (April 2019- March 2020) | Once monthly in triplicates |
| Omonijo et al. (26) | Retrospective | Nigeria | 2011 | Nigerian Meteorological Agency | Rainfall,  minimum and maximum air temperature,  relative humidity and wind speed | 10 years (1996- 2006) | Once monthly |
| Abbas et al. (13) | Retrospective | Nigeria | 2021 | Nigerian  Meteorological Agency | Temperature, wind, rain and humidity | 1 year (January 2016-January 2017) | - |
| Desalu, O. O (15) | Retrospective | Nigeria | 2011 | Wet (April-October) and dry (November- March) season | - | 3 years (November 2006-October 2009) | - |
| Narh et al. (27) | Retrospective | Ghana | 2021 | Wet (May- September) and dry (October- April) season | - | 6 years (2012- 2017) | - |
| Désiré et al. (9) | Retrospective | Cameroon | 2015 | Regional source of meteorology | Temperature and rainfall | 8 years (2007- 2014) | Once monthly |

**Table 9.** Summary of study findings on the associations between environmental exposures and the severity of obstructive lung diseases.

| Environmental exposures | Diseases | | | Severity of disease | |
| --- | --- | --- | --- | --- | --- |
|  | Asthma | Chronic bronchitis | Emphysema | COPD | Asthma |
| Temperature | (∅): (14)*, (6)  (+): (9) | (∅): (6) | (∅): (6) |  | (-): (13) |
| Humidity | (-): (14)  (+): (14)**, (26)* |  |  |  | (∅): (13) |
| Precipitation | (+): (9,26) |  |  |  | (∅): (13) |
| Wind speed |  |  |  |  | (∅): (13) |
| Wet season | (∅): (15) |  |  |  |  |
| Dry season |  |  |  | (+): (15) | (+): (27) |

(14): in dry season, (14)*: in dry and wet season, (14)**: in wet season, (26): in humid and savanna areas, (26)*: in humid areas

**Table 10.** Study findings regarding interventions that enhance knowledge about obstructive lung diseases and their risk factors

| Author (study) | Intervention | Description of intervention | Outcome |
| --- | --- | --- | --- |
| Jones et al. (41) | A train-the-trainer lung health educational program | - Training system: HCW 🡪 other HCW 🡪 community health workers 🡪 general population. - Educational materials: lung health and risk factors for lung damage (posters, flip-over charts, brochures, etc.) - Broadcasting of educational messages on local radio | - Knowledge of dangers of tobacco smoke post-campaign: high (85-95%) - Knowledge of dangers of biomass smoke post-campaign: Low (30-58%) |
| Brakema et al. (42) | Train-the-trainer educational program translated into other settings | See Description of (41) | -Knowledge: Significant increase in correct answers (Kyrgyzstan: 53 🡪 91%, Vietnam: 74 🡪 83%) -Higher acceptability and adequate use of improved single and multiple pot stoves instead of open fire |
| Nantanda et al. (43) | Health education sessions on biomass smoke | Educational sessions on biomass smoke given by midwives to pregnant women (posters, flip charts, leaflets) | - 45% increase in the knowledge of biomass smoke as risk factor for asthma. - Increased motivation to make changes: reducing smoke emissions by using dry wood and improving kitchen ventilation |
| Robertson et al. (40) | COPD-specific education package | COPD knowledge questionnaire (COPD-KQ) developed by an expert team and administered to COPD patients by a CHW before and 3 months after delivery of the education package using flipcharts (pathophysiology, diagnosis guidelines, management, and prevention) | - Significant improvement in COPD-KQ score (8 to 10.2 from pre- to post-education) - At baseline, knowledge was highest for shortness of breath as a symptom of COPD, biomass smoke as a cause of COPD and spirometry for diagnosis of COPD |

**Table 11.** Study findings regarding interventions that enhance disease control and adherence to treatment and/or behavioral changes

| Author (study) | Intervention | Description of intervention | Outcome |
| --- | --- | --- | --- |
| Siddharthan et al. (44) | Screening questionnaires to identify individuals with COPD in LMIC | Three screening questionnaires were assessed for their discriminative accuracy to identify individuals with COPD who will require further spirometry testing | - Participants identified with COPD had severe obstruction and were unaware of the diagnosis despite low quality of life - Participants with false positive results were likely to exhibit a history of asthma or chronic bronchitis |
| Cartwright et al. (45) | Health education sessions on biomass smoke | Long-term impact of education sessions of (43) | - Changes were made regarding the cooking area, the kitchen ventilation, the children’s proximity to smoke while cooking and the usage of solar power instead of candles for lighting - Cost-effectiveness (decreased costs of firewood buying and medical care due to smoke exposure) |
| Rylance et al. (46) | Enhanced asthma care package | - Intervention delivered by non-physicians: clinical assessment, inhaled treatment optimization and individualized asthma education   Control group received standard care from physicians | - Childhood Asthma Control Test (cACT) score at 3 months: 22.9 and 20.8 in the intervention and control group respectively - School absence and emergency health facility attendance was reported less in the intervention group - Self-reported ICS use was higher in the intervention group |
| Amorha et al. (47) | Pharmacist-led asthma education | - Intervention delivered either to the individual or the caregiver: asthma triggers and control, types of inhalers and handling of asthma attacks (during clinic visits and through phone calls and text messages)   Control group received the standard care of the hospital | - Significant improvement in the Asthma Control Test (ACT) score and the 8-item Morisky Medication Adherence Scale (MMAS- 8-Item) score in the individual intervention group compared to the control group at 3 and 6 months |
| Sooriyakanthan et al. (48) | Pulmonary rehabilitation sessions using limited resources | - Supervised sessions were conducted twice weekly for 6 weeks among the intervention group and included stretching exercises, walking and strength training exercises   Daily training walks and strength training exercises done twice weekly (using 2 bottles filled with enough water to equal the weight of the dumbbells) were instructed to complete at home | - It is safe and feasible to establish a pulmonary rehabilitation program using limited resources - Among the intervention group:  1. Chronic COPD questionnaire (CCQ) and COPD Assessment Test (CAT) improved at least four times the Minimum Clinically Important Difference (MCID) 2. MRC dyspnea scale improved more than the MCID  - Exercise performance using the 6-minute walk test (6MWD) and the incremental shuttle walk test (ISWD) was close to or better than the MCID |

**Table 12.** Study findings regarding improved cookstoves as an intervention method

| Author (study) | Intervention | Description of intervention | Outcome |
| --- | --- | --- | --- |
| Van Gemert et al. (49) | Implementation of different improved cookstoves/ heaters within the different settings | Improved cookstoves/heaters were introduced after understanding the setting’s environment and the heating and cooking behavior. In each setting, participants were able to choose between different cookstoves depending on convenience and availability of fuels | - Significant decrease in PM2.5 values post-intervention - Significant decrease in respiratory symptoms and school absence mainly in Uganda and Kyrgyzstan. - A high acceptance of the improved cookstoves/heaters was shown |
| Rennert et al. (50) | Implementation of smokeless cookstoves equipped with an improved combustion chamber and a chimney | Improved cookstoves were introduced to households where members received instructions on the use and maintenance of the stove | - Overall PEFR improved from 88.2% to 102.9% after the installation of smokeless stoves. - No significant change in subjective health status (cough or clinic visit) after the intervention |
| Critchley et al. (51) | Implementation of energy-efficient stoves equipped with a chimney to carry smoke outside of the house | Less wood was required when using the newly implemented stoves | - No difference in air quality post-intervention - Women reported fewer respiratory illnesses and improved spirometry tests post-intervention - Less money spent for extra fuel and less time wasted in wood collection |
| Rylance et al. (52) | Implementation of cleaner burning biomass-fueled cookstoves equipped with a solar panel to charge the stove-fan battery and a user training | Lung function findings and air pollutant exposure were monitored for 3 years to check for the various determinants of lung function including the effect of introducing two cleaner-burning biomass-fueled cookstoves | - Self-reported respiratory symptoms increased over the course of the study (13.6% to 36.2%) - Access to a cookstove was associated with decreased PM2.5. - Access to a cookstove did not improve neither FEV1 nor FVC. |

**References (studies included in the scoping review):**

1. Eghomwanre AF, Oguntoke O. Concentrations of indoor gaseous air pollutants and risk factors associated with childhood asthma in Benin City, Nigeria. Environmental Monitoring and Assessment [Internet]. 2022;194(5). Available from: https://www.embase.com/search/results?subaction=viewrecord&id=L2015880881&from=export

2. Munyai O, Nunu WN. Health effects associated with proximity to waste collection points in Beitbridge Municipality, Zimbabwe. Waste Management. 2020;105:501–10.

3. Akpeimeh GF, Fletcher LA, Evans BE. Exposure to bioaerosols at open dumpsites: A case study of bioaerosols exposure from activities at Olusosun open dumpsite, Lagos Nigeria. Waste Management. 2019;89:37–47.

4. Obaseki DO, Awopeju OF, Awokola BI, Adeniyi BO, Adefuye BO, Ozoh OB, et al. Domestic solid fuel combustion in an adult population in Nigeria: A cross sectional analysis of association with respiratory symptoms, quality of life and lung function. Respiratory Medicine. 2017;130:61–8.

5. Obiebi IP, Ibekwe RU, Eze GU. Lung function impairment among charcoal workers in an informal occupational setting in Southern Nigeria. African Journal of Respiratory Medicine. 2017;13(1):8–13.

6. Ayaaba E, Li Y, Yuan J, Ni C. Occupational respiratory diseases of miners from two gold mines in Ghana. International Journal of Environmental Research and Public Health [Internet]. 2017;14(3). Available from: https://www.embase.com/search/results?subaction=viewrecord&id=L614950682&from=export

7. Hamatui N, Beynon C. Particulate matter and respiratory symptoms among adults living in Windhoek, Namibia: A cross sectional descriptive study. International Journal of Environmental Research and Public Health [Internet]. 2017;14(2). Available from: https://www.embase.com/search/results?subaction=viewrecord&id=L614218213&from=export

8. Agrawal S. Effect of indoor air pollution from biomass and solid fuel combustion on prevalence of self-reported asthma among adult men and women in India: findings from a nationwide large-scale cross-sectional survey. Journal of Asthma. 2012;49(4):355–65.

9. Landry MMJ, Soleil BRA. The influence of climate change/variability on the prevalence of respiratory diseases: The case of asthma in Bamenda.

10. Pathak U, Gupta N, Suri J, Tyagi C. Effects of Biomass Fuel Generated Indoor Air Pollution on Prevalence of Self-reported Asthma among Adult Women in North Indian Rural Villages. 2018;

11. Mustapha BA, Blangiardo M, Briggs DJ, Hansell AL. Traffic air pollution and other risk factors for respiratory illness in schoolchildren in the niger-delta region of Nigeria. Environmental health perspectives. 2011;119(10):1478–82.

12. René KAK, Kouadio K, Siele S, Harvey AT, M’begnan C, Leandre K, et al. Prevalence of asthma in children under 5 years old exposed to air pollution in Abidjan,(Côte D’ivoire). International Journal of Recent Scientific Research Vol 10, Issue, 07 (A), pp 33353-33358, July, 2019. 2019;

13. Abbas A, Okpapi JU, Njoku CH, Abba AA, Isezuo SA, Danasabe IM. Influence of seasonal changes on asthma exacerbation in a sudan savanna region: An analysis of 87 cases. Annals of African medicine. 2021 Oct;20(4):302–6.

14. Eghomwanre AF, Oguntoke O, Taiwo AM. Levels of indoor particulate matter and association with asthma in children in Benin City, Nigeria. Environmental monitoring and assessment. 2022 Jun;194(7):467.

15. Desalu OO. Seasonal variation in hospitalisation for respiratory diseases in the tropical rain forest of South Western Nigeria. The Nigerian postgraduate medical journal. 2011 Mar;18(1):39–43.

16. McElroy S, Dimitrova A, Evan A, Benmarhnia T. Saharan Dust and Childhood Respiratory Symptoms in Benin. International Journal of Environmental Research and Public Health [Internet]. 2022;19(8). Available from: https://www.scopus.com/inward/record.uri?eid=2-s2.0-85128231468&doi=10.3390%2fijerph19084743&partnerID=40&md5=861d9173386683015e36283a05ba153b

17. Oyebanji FF, Ana GREE, Mijinyawa Y, Ogunseye OO. Predicting exposure to dust particles using spirometric index and perception studies among farmers in selected farm settlements in ogun state, nigeria. Aerosol and Air Quality Research [Internet]. 2021;21(7). Available from: https://www.scopus.com/inward/record.uri?eid=2-s2.0-85110021488&doi=10.4209%2faaqr.200509&partnerID=40&md5=8150d24f243690d973008c9128682ae1

18. Woolley KE, Bagambe T, Singh A, Avis WR, Kabera T, Weldetinsae A, et al. Investigating the association between wood and charcoal domestic cooking, respiratory symptoms and acute respiratory infections among children aged under 5 years in uganda: A cross-sectional analysis of the 2016 demographic and health survey. International Journal of Environmental Research and Public Health. 2020;17(11):1–14.

19. Tamire M, Addissie A, Kumie A, Husmark E, Skovbjerg S, Andersson R, et al. Respiratory symptoms and lung function among Ethiopian women in relation to household fuel use. International Journal of Environmental Research and Public Health [Internet]. 2020;17(1). Available from: https://www.scopus.com/inward/record.uri?eid=2-s2.0-85076969738&doi=10.3390%2fijerph17010041&partnerID=40&md5=91a65539889c39b16436ffa00be17597

20. Brakema EA, Tabyshova A, Kasteleyn MJ, Molendijk E, Van Der Kleij RMJJ, Van Boven JFM, et al. High COPD prevalence at high altitude: Does household air pollution play a role? European Respiratory Journal [Internet]. 2019;53(2). Available from: https://www.scopus.com/inward/record.uri?eid=2-s2.0-85061435100&doi=10.1183%2f13993003.01193-2018&partnerID=40&md5=abfb719a75d40feb279ce2602ddb3d05

21. Siddharthan T, Grigsby MR, Goodman D, Chowdhury M, Rubinstein A, Irazola V, et al. Association between household air pollution exposure and chronic obstructive pulmonary disease outcomes in 13 low- and middle-income country settings. American Journal of Respiratory and Critical Care Medicine. 2018;197(5):611–20.

22. Das I, Jagger P, Yeatts K. Biomass Cooking Fuels and Health Outcomes for Women in Malawi. EcoHealth. 2017;14(1):7–19.

23. Ngahane BHM, Ze EA, Chebu C, Mapoure NY, Temfack E, Nganda M, et al. Effects of cooking fuel smoke on respiratory symptoms and lung function in semi-rural women in Cameroon. International Journal of Occupational and Environmental Health. 2015;21(1):61–5.

24. Mohammad Y, Shaaban R, Al-Zahab BA, Khaltaev N, Bousquet J, Dubaybo B. Impact of active and passive smoking as risk factors for asthma and COPD in women presenting to primary care in Syria: First report by the WHO-GARD survey group. International Journal of COPD. 2013;8:473–82.

25. Quansah R, Bend JR, Armah FA, Bonney F, Aseidu J, Yawson DO, et al. Respiratory and non-respiratory symptoms associated with pesticide management practices among farmers in Ghana’s most important vegetable hub. Environ Monit Assess. 2019 Nov 4;191(12):716.

26. Omonijo AG, Oguntoke O, Matzarakis A, C.O. A. A Study of Weather Related Respiratory Diseases in Eco-climatic Zones. African Physical Review. 2011 Jan 1;5:41–56.

27. Narh CT, Der JB, Afetor M, Ofosu A, Blettner M, Wollschlaeger D. Sociodemographic factors associated with time to discharge for hospitalised patients with asthma and asthma exacerbation using the Ghana Health Service District Information Management System 2 (DHIMS-2) database, 2012-2017. BMJ Open Respir Res. 2021 Nov;8(1):e001034.

28. Olujimi OO, Ana GREE, Ogunseye OO, Fabunmi VT. Air quality index from charcoal production sites, carboxyheamoglobin and lung function among occupationally exposed charcoal workers in South Western Nigeria. SpringerPlus. 2016 Sep 13;5(1):1546.

29. Subramaniam S, Ganesan A, Raju N, Prakash C. Investigation of indoor air quality and pulmonary function status among power loom industry workers in Tamil Nadu, South India. Air Qual Atmos Health [Internet]. 2023 Sep 30 [cited 2024 Jan 2]; Available from: https://doi.org/10.1007/s11869-023-01439-5

30. Oo TW, Thandar M, Htun YM, Soe PP, Lwin TZ, Tun KM, et al. Assessment of respiratory dust exposure and lung functions among workers in textile mill (Thamine), Myanmar: a cross-sectional study. BMC Public Health. 2021 Apr 7;21(1):673.

31. Leon-Kabamba N, Ngatu NR, Kakoma SJB, Nyembo C, Mbelambela EP, Moribe RJ, et al. Respiratory health of dust-exposed Congolese coltan miners. Int Arch Occup Environ Health. 2018 Oct 1;91(7):859–64.

32. Dushyant K, Walia GK, Devasenapathy N. Lung Function and Respiratory Morbidity Among Informal Workers Exposed to Cement Dust: A Comparative Cross-Sectional Study. 2023 Jul 4;89(1):47.

33. Jestin-Guyon N, Ouaalaya EH, Harison MT, Ravahatra K, Rakotomizao J, Raharimanana RN, et al. Impact of biomass fuel smoke on respiratory health of children under 15 years old in Madagascar. Respiratory Medicine and Research. 2023 Jun 1;83:100989.

34. Meme H, Amukoye E, Bowyer C, Chakaya J, Das D, Dobson R, et al. Asthma symptoms, spirometry and air pollution exposure in schoolchildren in an informal settlement and an affluent area of Nairobi, Kenya. Thorax. 2023 Nov;78(11):1118–25.

35. Wafula ST, Nalugya A, Mendoza H, Kansiime WK, Ssekamatte T, Walekhwa AW, et al. Indoor air pollutants and respiratory symptoms among residents of an informal urban settlement in Uganda: A cross-sectional study. PLOS ONE. 2023 Aug 17;18(8):e0290170.

36. Zoller T, Mfinanga EH, Zumba TB, Asilia PJ, Mutabazi EM, Wimmersberger D, et al. Symptoms and functional limitations related to respiratory health and carbon monoxide poisoning in Tanzania: a cross sectional study. Environmental Health: A Global Access Science Source [Internet]. 2022;21(1). Available from: https://www.scopus.com/inward/record.uri?eid=2-s2.0-85127492935&doi=10.1186%2fs12940-022-00847-x&partnerID=40&md5=1c93ffb5821ea3086dd81cd8c854e626

37. Tamire M, Worku A, Addissie A, Ayele S, Haile T, Bekele A. Blinded to Their Own Daily Sufferings: The Lived Experience of Patients with Chronic Obstructive Pulmonary Disease in Addis Ababa. Ethiopian Journal of Health Development [Internet]. 2021;35(4). Available from: https://www.scopus.com/inward/record.uri?eid=2-s2.0-85134368836&partnerID=40&md5=544a05282d325fcc2a6cab9057e2f22d

38. Brakema EA, Tabyshova A, Van Der Kleij RMJJ, Sooronbaev T, Lionis C, Anastasaki M, et al. The socioeconomic burden of chronic lung disease in low-resource settings across the globe - An observational FRESH AIR study. Respiratory Research [Internet]. 2019;20(1). Available from: https://www.scopus.com/inward/record.uri?eid=2-s2.0-85077153352&doi=10.1186%2fs12931-019-1255-z&partnerID=40&md5=876a828208a6f31ec122903b96db6226

39. Egere U, Shayo EH, Chinouya M, Taegtmeyer M, Ardrey J, Mpagama S, et al. “Honestly, this problem has affected me a lot”: a qualitative exploration of the lived experiences of people with chronic respiratory disease in Sudan and Tanzania. BMC Public Health. 2023 Mar 13;23(1):485.

40. Robertson NM, Siddharthan T, Pollard SL, Alupo P, Flores-Flores O, Rykiel NA, et al. Development and Validity Assessment of a Chronic Obstructive Pulmonary Disease Knowledge Questionnaire in Low- and Middle-Income Countries. Ann Am Thorac Soc. 2021 Aug;18(8):1298–305.

41. Jones R, Kirenga B, Buteme S, Williams S, Van Gemert F. A novel lung health programme addressing awareness and behaviour-change aiming to prevent chronic lung diseases in rural Uganda. African Journal of Respiratory Medicine. 2020;14(2):2–9.

42. Brakema EA, van Gemert FA, Williams S, Sooronbaev T, Emilov B, Mademilov M, et al. Implementing a context-driven awareness programme addressing household air pollution and tobacco: a FRESH AIR study. NPJ primary care respiratory medicine. 2020 Oct;30(1):42.

43. Nantanda R, Buteme S, van Kampen S, Cartwright L, Pooler J, Barton A, et al. Feasibility and acceptability of a midwife-led health education strategy to reduce exposure to biomass smoke among pregnant women in Uganda, A FRESH AIR project. Global Public Health. 2019;14(12):1770–83.

44. Siddharthan T, Pollard SL, Quaderi SA, Rykiel NA, Wosu AC, Alupo P, et al. Discriminative Accuracy of Chronic Obstructive Pulmonary Disease Screening Instruments in 3 Low- and Middle-Income Country Settings. JAMA. 2022 Jan;327(2):151–60.

45. Cartwright LL, Callaghan LE, Jones RC, Nantanda R, Fullam J. Perceptions of long term impact and change following a midwife led biomass smoke education program for mothers in rural Uganda: A qualitative study. Rural and Remote Health [Internet]. 2022;22(1). Available from: https://www.scopus.com/inward/record.uri?eid=2-s2.0-85124776081&doi=10.22605%2fRRH6893&partnerID=40&md5=2e59244ff84fb86eb6885710eaeb1f06

46. Rylance S, Chinoko B, Mnesa B, Jewell C, Grigg J, Mortimer K. An enhanced care package to improve asthma management in Malawian children: a randomised controlled trial. Thorax. 2021 May;76(5):434–40.

47. Amorha KC, Okonta MJ, Ukwe CV. Impact of pharmacist-led educational interventions on asthma control and adherence: single-blind, randomised clinical trial. Int J Clin Pharm. 2021 Jun;43(3):689–97.

48. Sooriyakanthan M, Orme MW, Sivapalan K, Selvaratnam G, Singh SJ, Wimalasekera S. A feasibility trial of pulmonary rehabilitation for patients with COPD in a low resource setting: Jaffna, Sri Lanka. BMC Pulmonary Medicine. 2022 Aug 8;22(1):302.

49. van Gemert F, de Jong C, Kirenga B, Musinguzi P, Buteme S, Sooronbaev T, et al. Effects and acceptability of implementing improved cookstoves and heaters to reduce household air pollution: a FRESH AIR study. npj Primary Care Respiratory Medicine [Internet]. 2019;29(1). Available from: https://www.scopus.com/inward/record.uri?eid=2-s2.0-85070997688&doi=10.1038%2fs41533-019-0144-8&partnerID=40&md5=45a23a34c3c631a7b964990febfa68f7

50. Rennert WP, Porras Blanco RM, Muniz GB. The effects of smokeless cookstoves on peak expiratory flow rates in rural Honduras. Journal of Public Health (United Kingdom). 2015;37(3):455–60.

51. Critchley K, Teather K, Hughes H, Macdonald A, Gibson M, J B, et al. Air quality, respiratory health and wood use for women converting from low- to high-efficiency stoves in rural Kenya. 23rd International Conference on Modelling, Monitoring and Management of Air Pollution, AIR 2015. 2015;198:205–16.

52. Rylance S, Jewell C, Naunje A, Mbalume F, Chetwood JD, Nightingale R, et al. Non-communicable respiratory disease and air pollution exposure in Malawi: a prospective cohort study. Thorax. 2020 Mar;75(3):220–6.
